# Disentangling neural processing of masked and masking stimulus by means of event-related contralateral – ipsilateral differences of EEG potentials

**DOI:** 10.2478/v10053-008-0025-0

**Published:** 2008-07-15

**Authors:** Rolf Verleger, Piotr Jaśkowski

**Affiliations:** 1 Department of Neurology, University of Lübeck, Germany; 2 Department of Cognitive Psychology, University of Finance and Management, Warszawa, Poland

**Keywords:** event-related potentials, masking, masked priming, N2pc, LRP, N2cc

## Abstract

In spite of the excellent temporal resolution of event-related EEG potentials (ERPs), the overlapping potentials evoked by masked and masking stimuli are hard to disentangle. However, when both masked and masking stimuli consist of pairs of relevant and irrelevant stimuli, one left and one right from fixation, with the side of the relevant element varying between pairs, effects of masked and masking stimuli can be distinguished by means of the contralateral preponderance of the potentials evoked by the relevant elements, because the relevant elements may independently change sides in masked and masking stimuli. Based on a reanalysis of data from which only selected contralateral-ipsilateral effects had been previously published, the present contribution will provide a more complete picture of the ERP effects in a masked-priming task. Indeed, effects evoked by masked primes and masking targets heavily overlapped in conventional ERPs and could be disentangled to a certain degree by contralateral-ipsilateral differences. Their major component, the N2pc, is interpreted as indicating preferential processing of stimuli matching the target template, which process can neither be identified with conscious perception nor with shifts of spatial attention. The measurements showed that the triggering of response preparation by the masked stimuli did not depend on their discriminability, and their priming effects on the processing of the following target stimuli were qualitatively different for stimulus identification and for response preparation. These results provide another piece of evidence for the independence of motor-related and perception-related effects of masked stimuli.

##  

The purpose of this contribution is to provide a picture of the strengths and limits of the use of event-related EEG potentials (ERPs) as a measure of brain activity in masked priming.

Taking previously published data of ours as an example (Jaśkowski, van der Lubbe, Schlotterbeck, & Verleger, 2002), we will provide a more complete overview of the data. It will become obvious what information can be obtained from conventional ERPs, and what additional information may be provided by focusing on differences between recording sites contralateral minus ipsilateral to the relevant stimulation.

On the occasion of this reanalysis, we will try to resolve an apparent paradox that emerged in these data for the major perceptual component of the contralateral-ipsilateral differences, the N2pc. (“N2pc” stands for negativity at posterior sites contralateral to the evoking stimulus in the time range of the N2, which is the 2^nd^ major negative peak of the event-related potential). The way to resolve the paradox might lead via a conceptual clarification of what process is indicated by N2pc.

The analysis will provide some more arguments for divergent effects of masked stimuli on perceptual identification and response priming. More generally, we will show by means of this analysis that ERPs recorded from the intact human scalp can provide valuable information about the time-course of processing in masked priming.

## EVENT-RELATED EEG POTENTIALS

When the neurons of the brain communicate with each other, voltage fluctuations arise within the medium that surrounds the receiving neurons (Birbaumer, Elbert, Canavan, & Rockstroh, 1990; Logothetis, Pauls, Augath, Trinath, & Oeltermann, 2001; Zschocke, 2002). Under favorable physical and geometric conditions, some part of these post-synaptic local-field potentials can be measured at the scalp as EEG (Lutzenberger, Elbert, & Rockstroh, 1987). Due to the abundance of neural activity, voltage fluctuations of different origins overlap at the scalp, so a convenient method to extract lawful regularities works by repeating homologous events and averaging EEG across trials, time-locked to the events. Thereby, event-related EEG potentials (ERPs) are obtained (Luck, 2005; Zani & Proverbio, 2002).

No other method of measuring effects of neuronal activity non-invasively has better temporal resolution than ERPs (Kutas & Federmeier, 1998). Therefore, recording ERPs is the most obvious method to learn more about brain processing of masked and masking stimuli: Due to their good temporal resolution, ERPs are expected to provide a chance to disentangle the brain responses to masked and masking stimuli although these stimuli are separated by only fractions of seconds.

## CAN ERPS DISENTANGLE EFFECTS OF MASKED AND MASKING EVENTS?

**Figure 1.**
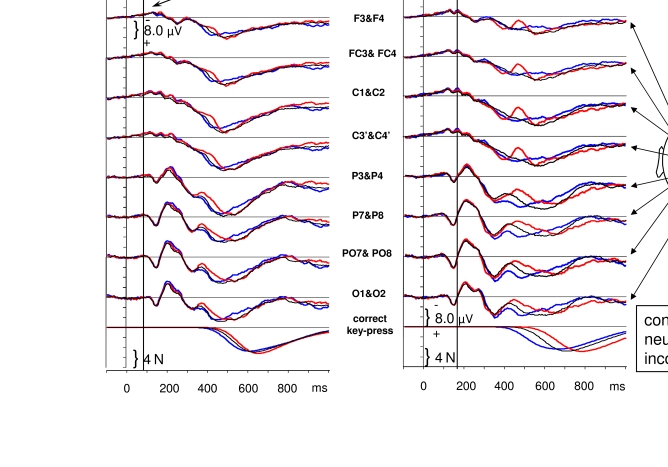
ERPs evoked by the sequence of primes and targets, from 100 ms before prime onset until 1 s afterwards. Grand means across 12 participants. Trials with 83 ms SOA between primes and targets are compiled in the left half, trials with 167 ms in the right half. “Congruent” means that the relevant shape was on the same side in primes as in targets, “incongruent” means different sides, “neutral” denotes two irrelevant shapes in the primes. Each panel displays waveshapes averaged across a pair of symmetrical left and right positions, from anterior sites of the scalp (top) to occipital sites (2nd panels from bottom), against a reference at the nose. The bottom panels display the time course of the forces exerted on the response keys.

**Figure 2.**
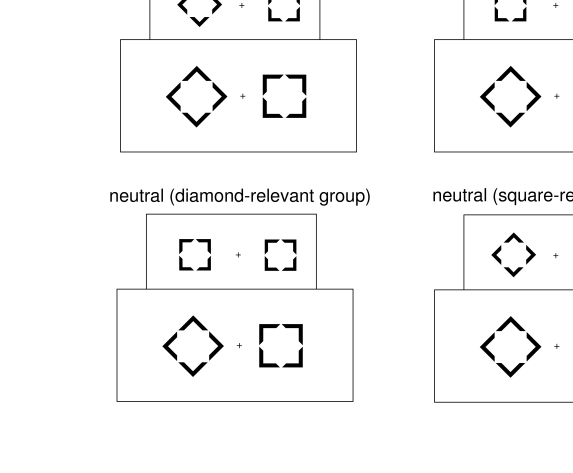
Possible sequences of primes and targets, exemplified for targets with diamond on the left and rectangle on the right, so for the diamond-relevant participants the correct response was to press the left key, and for the rectangle-relevant participants to press the right key. Primes (smaller shapes) were presented for 17 ms, SOAs between primes and targets were 83 ms or 167 ms, targets were presented for 100 ms.

In fact, ERPs evoked by pairs of masked and masking stimuli will not easily disentangle. This is illustrated in Figure 1. (These data were recorded in Experiment 1 of Jaśkowski, van der Lubbe, Schlotterbeck, & Verleger, 2002, but were not reported in that publication.) In this experiment, both masked and masking stimuli were squares or diamonds (Figure 2), with the outer outlines of the smaller masked stimuli fitting the inner outlines of the masking stimuli, thus being subject to masking by metacontrast. A full account of the experimental methods is provided in the Appendix. ERPs will be reported in this paper from the choice-response part of the experiment. In this part, the masking stimuli were the “targets” to which a manual response had to be made, and the preceding masked stimuli were “primes” because they were expected to affect the manual response to the following target. Participants had to press the left or right key depending on the side of the relevant shape in the target stimulus. (The relevant shape was the diamond for half of the participants, and the square for the other half.) Primes could be congruent, incongruent, or neutral in their relation to the following target, that is, the relevant shape could be on the same side as in the target, on the opposite side, or no relevant shape was included in the prime. Stimulus-onset asynchronies (SOA) between primes and targets were either 83 ms or 167 ms (henceforth *SOA83* and *SOA167*). Prime-target congruence and SOA were randomly varied across trials. The rationale of the SOA variation was to use *SOA83* as the condition where primes were indistinguishable and *SOA167* as a control condition where primes were still hard to distinguish but above the “threshold” of awareness.

Figure 1 provides an overview of the ERP results. Time point 0 is the onset of the primes. Depicted are the grand-average voltage fluctuations across the 12 participants, recorded from several scalp sites, separately for the two SOAs and the three prime-target congruence relations. The time point of overt responses can be seen in the same waveshape format as the ERPs in the bottom panels where the grand averages of the output voltages of the force-sensitive response keys are depicted. Forces that exceeded 2 N were counted as responses. Mean response times in congruent, neutral, incongruent trials were 376, 394, 414 ms with *SOA83*, and 251, 311, 379 ms with *SOA167*. (Since the x-axis in Figure 1 is related to prime onset, these times translate to 459, 477, 497 ms with *SOA83*, and 418, 478, 546 ms with *SOA167* in Fig. 1.) These effects of congruence were significant with both SOAs and significantly larger with *SOA167* than with *SOA83* (Jaśkowski et al., 2002).

The first obvious evoked response started at about 100 ms after prime onset at posterior sites, including the typical components of the visual evoked response: the positive P1 (plotted downwards) and the following negative N1 (plotted upwards). Of interest would be: first, to have a clear distinction between components evoked by primes from components evoked by targets, second, to see effects of prime-target congruence (i.e., differences between the three line types within any panel) in the components evoked by the target. That distinction and those effects would be of most interest if they were related to perceptual processes, that is, if they occurred early in time, before overt responding, and at posterior sites, recorded from scalp sites above the visual cortex.

### Early effects at posterior sites

**Figure 3.**
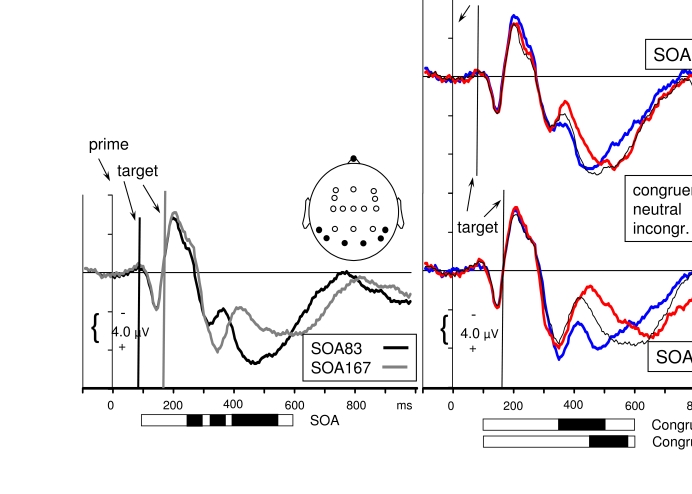
Waveshapes pooled across the posterior sites of the head (P7, P8, PO7, PO8, O1, O2, i.e., across 2^nd^ to 4^th^ panels from bottom in Figure 1; positions are indicated by the black dots in the schematic head). The waveshapes in the right panels are the same as in Figure 1 (except for pooling across P, PO, O and greater scale).The waveshapes in the left panel have been additionally pooled across congruent, neutral, incongruent, to focus on main effects of targets which are obtained by comparing *SOA83* (black) with *SOA167* (grey).Horizontal bars, extending from 100 ms to 600 ms display significant effects of ANOVAs performed on 25 ms intervals between 100 ms and 600 ms after prime onset. Black shading indicates *p*<.05.

Therefore, Figure 3 displays with better resolution the visually evoked potentials recorded at posterior sites (pooled across P7, P8, PO7, PO8, O1, O2). The left panel of Fig. 3 highlights the effects of SOA, by comparing *SOA83* to *SOA167*, pooling across congruent, neutral, and incongruent trials. The right panels show (like Fig.1) the separate waveshapes of congruent, neutral, and incongruent trials. To analyze these data, mean amplitudes were formed for intervals of 25 ms duration, beginning with 105-125 ms and ending with 575-600 ms. Analyses of Variance (ANOVAs) were conducted on each interval, with the factors Hemisphere (P7, PO7, O1 vs. P8, PO8, O2, i.e., left vs. right), SOA (83 / 167), Congruence (congruent, neutral, incongruent). In this paragraph, the effects from 105 ms to 350 ms will be discussed, which interval forms the time-range of the P1, N1, and P2 components of the visually evoked potential.

The first visible component, the positive P1, was obviously evoked by the prime only, first because with *SOA167* it reached its peak even before the target was presented, second because it had a stable latency with respect to prime onset (at 145 ms), third because there were no effects of SOA and Congruence from 105 ms to 200 ms in the ANOVAs on 25 ms intervals. This time range included the ascending slope of the following N1 component which, peaking at 205 ms, likewise was obviously evoked by the prime only. The upper right panel of Fig. 3 suggests some effect of Congruence at 205-225 ms at *SOA83*. However, this effect, which looks like an enhanced N1 with congruent primes, did not reach significance.^1^

The main effect of SOA first became significant at 255-275 ms, with more negativity at *SOA167* than at *SOA83* on the descending slope of the N1. This might be interpreted as an N1 evoked by the target at *SOA167*, and indeed the recordings from O1 and O2 (Figure 1) provide a cogent impression of a second negative peak at this latency, following the first negative peak at 205 ms. On the other hand, the latency of this component is just about 100 ms after target onset (265-167 ms), which is much earlier than the 200 ms latency of the N1 evoked by the prime. Alternatively, this greater negativity at *SOA167* might rather be due to greater positivity at *SOA83*, perhaps caused by the P1 component evoked by the *SOA83* target. But this P1 would be delayed, having a latency of about 180 ms (265-83 ms). In fact, other data suggest the first alternative, that sequences of consecutive stimuli evoke continuous N1-type negative potentials (Verleger, Jaśkowski, & Wascher, 2005). The major point to make from these considerations is that it is actually difficult to see an independent visual potential evoked by the second stimulus in a series.

Possible reasons for this difficulty include the speculations that the P1-N1 complex is most sensitive to sudden onsets, and therefore is subject to habituation (but see Sable, Low, Maclin, Fabiani, & Gratton, 2004, for a concise discussion of the effect of top-down factors on alleged habituation in the case of auditory stimuli) and that the P1-N1 complex consists of alpha oscillations that are reset to phase by the first event (Hanslmayr, Klimesch, Sauseng, Gruber, Doppelmayr, Freunberger et al., 2007; Makeig, Westerfield, Jung, Enghoff, Townsend, Courchesne, et al., 2002; critically: Yeung, Bogacz, Holroyd, & Cohen, 2004) and cannot be reset again by the second event.^2^

In spite of the unclear separation of components evoked by the first and the second stimulus, there might still have been differential effects due to congruence between prime and target. However, there was no effect of Congruence at *p* < .05 or better before 350 ms (see below), that is, before the turning point of the positivity (“P2”) following the P1-N1 complex.

To summarize, the ERP data recorded from posterior sites in the first 350 ms after prime onset do not allow for a clear separation between perceptual components evoked by the target from components evoked by the prime. Nor were there effects of prime-target congruence.

### Later effects

**Figure 4.**
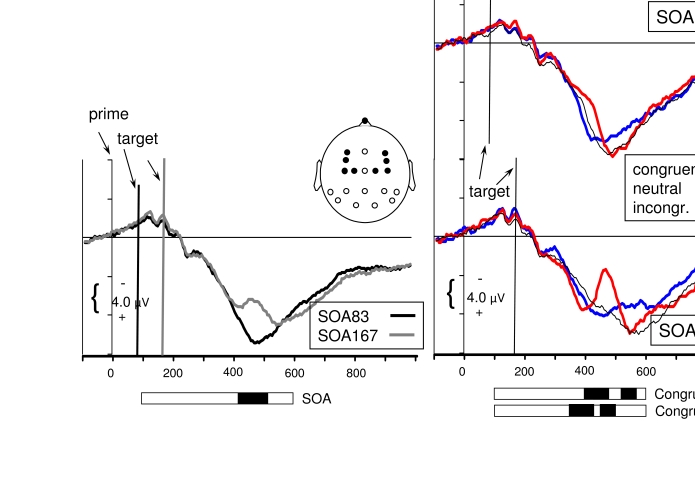
Waveshapes pooled across the anterior sites of the head (F3, F4, FC3, FC4, C1, C2, C3’, C4, i.e., across 1st to 4th panels from top in Figure 1; positions are indicated by the black dots in the schematic head). The waveshapes in the right panels are the same as in Figure 1 (except for pooling across F, FC, C and greater scale). The waveshapes in the left panel have been additionally pooled across congruent, neutral, incongruent, to focus on main effects of targets which are obtained by comparing *SOA83* (black) with *SOA167* (grey). Horizontal bars, extending from 100 ms to 600 ms display significant effects of ANOVAs performed on 25 ms intervals between 100 ms and 600 ms after prime onset. Black shading indicates *p*<.05.

Inspection of Figure 1 suggests distinct effects of prime-target congruence at later latencies, different for anterior and posterior recording sites. ANOVAs on mean amplitudes of 25 ms intervals were therefore also done for pooled values of anterior recordings (lateral F, FC, C sites: F3, F4, FC3, FC4, C3, C4, C1, C2). Like Figure 3 did for posterior sites, Figure 4 displays with better resolution these pooled potentials recorded at anterior sites.

At these anterior sites, a negative component, “N2”, was specifically evoked by incongruent prime-target sequences: 380-450 ms with *SOA83*, 430-500 ms with *SOA167*, as indicated by effects of Congruence or effects of Congruence x SOA (right panels of Figure 4). Neutral and congruent prime-target sequences did not differ from each other. The anterior N2 is the typical response to a mismatch of visual stimuli (Wang, Tian, Wang, Cui, Zhang, & Zhang, 2003; Wang, Cui, Wang, Tian, & Zhang, 2004), often interpreted as inhibition of a tendency to respond inappropriately (Kok, 1986; Kopp, Mattler, Goertz, & Rist, 1996; Kopp, Rist, & Mattler, 1996) or more basically as detection of conflict (Donkers & van Boxtel, 2004). This component, starting 260-300 ms after target onset (380 minus 83 ms with *SOA83*, 430 minus 167 ms with *SOA167*), was the first measurable brain response to prime-target incongruence in the present analysis. Importantly, this component was evoked by incongruent prime-target sequences even if primes were not consciously distinguishable, at *SOA83*. Moreover, surprisingly from first glance at Figure 1, the ANOVA indicated this effect to be not smaller with *SOA83* than with *SOA167*, as indicated by the lack of a Congruence x SOA interaction, F(2,22) = 0.5, n.s., when intervals of maximum N2 amplitude were compared to each other, 405-425 ms with *SOA83* vs. 455-475 ms with *SOA167*. The difference between incongruent and congruent sequences amounted to -3.6 µV with *SOA83* and to -4.5 µV with *SOA167*. The apparent difference of the congruence effects between both SOAs was modeled by ANOVA as a main effect of SOA 430-525 ms (left panel of Figure 4), with generally more negative values with *SOA167* than with *SOA83*.

Later in time (530-575 ms) congruent prime-target sequences produced more negative amplitudes than both neutral and incongruent sequences, which did not differ from each other. Interpretation of this effect is somewhat unclear. Possibly, processing of congruent sequences came to its end earlier than in the other cases, causing the waveshape to start returning to the baseline.

By its lacking dependence on awareness, the anterior N2 calls to mind auditory mismatch negativity (Näätänen & Winkler, 1999). However, analogues of MMN in the visual modality have their focus at posterior sites, specific to the visual modality, and occur earlier than N2 (Pazo-Alvarez, Cadaveira, & Amenedo, 2003). Another link may be drawn to Ne, the error-related negativity, which may also reflect response conflict and was shown to be independent of error awareness (Belopolsky & Kramer, 2006; Nieuwenhuis, Ridderinkhof, Blom, Band, & Kok, 2001). Ne is presumably generated in the rostral portion of the anterior cingulate cortex (Debener, Ullsperger, Siegel, Fiehler, von Cramon, & Engel, 2005). There might indeed be similar mechanisms involved in generating N2 and Ne although these two components are certainly not identical (e.g., Bartholow, Pearson, Dickter, Sher, Fabiani, & Gratton, 2005).

At posterior sites (Figure 3), effects of SOA from 330 ms to 375 ms reflected a posterior N2 with *SOA83*, with its peak at about 350 ms, followed by effects of SOA from 380 ms to 550 ms that reflected the posterior N2 with *SOA167*, peaking at about 420 ms, as well as the ensuing smaller P3 with *SOA167*. Effects of congruence started even earlier than at anterior sites and behaved differently: From 355 ms until 400 ms, that is, at the descending slope of the N2 with *SOA83* and at the ascending slope of the N2 with *SOA167*, both neutral and incongruent primes led to more negative waveshapes than did congruent primes, indistinguishably for *SOA83* and *SOA167*. From 405 ms to 500 ms, incongruent primes additionally led to more negative waveshapes than neutral primes, and this effect was reliably larger with *SOA167* (statistically distinguishable from *SOA83* from 455 ms onwards, where effects ceased to exist with *SOA83*). At least this latter effect, encompassing the time range of the P3 component and reflecting the different delays of the P3 in neutral and incongruent conditions, seemed to be closely related to overt responding, faithfully reflecting the differences in response times, which is to be expected because P3 latency reflects changes of response times whenever responses are fast (Verleger, 1997), forming the link from stimulus processing to response execution (Verleger, Görgen, Jaśkowski, 2005). The earlier effect (enhanced negativity of neutral and incongruent sequences 355-400 ms, roughly 250 ms after target onset) might be more interesting, possibly reflecting perceptual registration of a mismatch (though too late to be classified as a visual mismatch negativity, which reaches its peak before 150 ms, cf. Czigler, Balász, & Winkler, 2002; Pazo-Alvarez, Cadaveira, & Amenedo, 2003; Winkler, Czigler, Sussman, Horváth, & Balász, 2005) but it cannot be excluded that this effect simply reflects the earlier start of response processing with congruent stimuli, which might have pushed the congruent waveshapes earlier into the positive direction.

### Summary of effects in conventional ERPs

The major unambiguous effect of congruence was the anterior N2, reflecting a process related to conflict detection, emerging about 260-350 ms after target onset. Of much interest, this effect was not reliably smaller when primes were indistinguishable (*SOA83* vs. *SOA167*), thus it possibly took place independently of conscious perception of the primes.

But at posterior sites, overlying the visual cortex, ERPs did not allow for a clear separation of target-evoked potentials from prime-evoked potentials. The later posterior effects that did arise as a function of prime-target congruence might indicate response-related effects that occurred as correlates of the differing response times, rather than indicating true perception-related effects.

## HOW TO DISENTANGLE EFFECTS OF MASKED AND MASKING EVENTS IN ERPS

With overlap of prime- and target-evoked visual potentials unavoidable, how can specific effects be found?

One approach taken by ERP research is to separately estimate the contributions of two adjacent stimuli by varying their SOAs over a number of different values, to provide enough variance, and then removing the effects of one stimulus from the other by a reciprocal iterative procedure (van der Lubbe & Woestenburg, 1999; Woldorff, 1993). However, at least the formal AdjAR approach by Woldorff (1993) presupposes that the ERPs evoked by the stimuli are principally constant across this SOA variation. This is, of course, problematic with masked stimuli, which may be unidentifiable with some SOAs and produce conscious perception at other SOAs. Further, the approach implies the practical problem that experimental sessions have to be extended in order to get good estimates of ERPs for each SOA.

Therefore, we took a different approach. One convenient way taken by ERP research is to tag a “marker” to the effect under study and then to isolate the marker by subtracting the condition without the marker from the condition with the marker. In principle, this is the same rationale as used in fMRI studies, where activation is compared between some experimental condition and some control condition, and when this subtraction would provide unclear results, some “marker” is used, for example, faces would be used in some critical condition, known to specifically activate the “fusiform face area” (Vuilleumier, Sagiv, Hazeltine, Poldrack, Swick, Rafal et al., 2001) or words would be used, known to activate areas specialized in reading (Rees, Russell, Frith, & Driver, 1999).

For example, in order to study the processing of the 2^nd^ target in the attentional-blink paradigm, Vogel and Luck (2002; see also Sessa, Luria, Verleger, & Dell’Acqua, 2007) presented the 2^nd^ target in only 20% of their trials. In this way, the 2^nd^ target became an infrequent event. Relevant infrequent events evoke a P3 component, therefore the P3 measured in the difference of averages (trials with 2^nd^ target minus trials with distractors only) could be safely interpreted as an effect evoked by the 2^nd^ target, with potentials evoked both by the 1st target and by the ongoing chain of distractors being subtracted out.

Even closer to perception, Deouell, Amihai, and Bentin (2006) presented faces and watches as masked targets. Faces are known to evoke a special component (“N170”; Carmel & Bentin, 2002; Gauthier, Curran, Curby, & Collins, 2003), therefore subtraction of watches from faces was expected to cancel components common to both stimuli as well as potentials evoked by the masks and to indicate whether there was any face-specific activation, in the absence of the participants’ ability to reliably distinguish between faces and watches.

In our approach the “marker” attached to make the potential unique was the side of the relevant shape. When shapes are simultaneously presented left and right from fixation and the relevant shape is on one side but not on the other, an “N2pc” is evoked: More negativity is recorded at the scalp above the visual cortex contralateral to the relevant shape than ipsilateral, with a peak at about 250 ms after stimulus onset (e.g., Eimer, 1996; Hopf, Luck, Boelmans, Schoenfeld, Boehler, Rieger, & Heinze, 2006; Luck & Hillyard, 1994; Wascher & Wauschkuhn, 1996; Wauschkuhn, Verleger, Wascher, Klostermann, Burk, Heide, & Kömpf, 1998). Applying this here leads to the expectation that by forming the difference between potentials at symmetrical sites, contralateral minus ipsilateral to the relevant shape, any components evoked by prime and targets that do not differ between sides will be cancelled, leaving for analysis the processing related to the difference between relevant and irrelevant shapes. Importantly, this is expected to hold true for the prime pair and for the target pair. Of course, these two differences will again overlap, as with conventional ERPs. However, when leaving constant the side of the relevant shape in the target, then by alternating the side of the relevant shape in the prime pair, the N2pc evoked by the prime is expected to change sides and should therefore disentangle from the N2pc evoked by the target.

**Figure 5.**
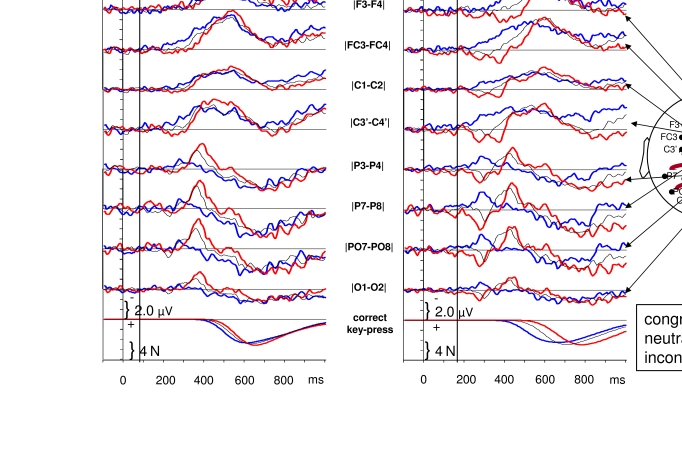
Contralateral-ipsilateral differences in ERPs evoked by the sequence of primes and targets, from 100 ms before prime onset until 1 s afterwards, with contralateral and ipsilateral defined with respect to side of the relevant element in the target, = side of the response. Grand means across 12 participants. Trials with 83 ms SOA between primes and targets are compiled in the left half, trials with 167 ms in the right half. “Congruent” means that the relevant shape was on the same side in primes as in targets, “incongruent” means different sides, “neutral” denotes two irrelevant shapes in the primes. Each panel displays difference waveshapes between a pair of symmetrical left and right positions, from anterior sites of the scalp (top) to occipital sites (2^nd^ panels from bottom). The bottom panels display the time course of the forces exerted on the response keys (identical to Figure 1).

Figure 5 displays the difference waveshapes between symmetrical scalp sites contralateral minus ipsilateral to the relevant shape in the target. In our first report of these data (Jaśkowski et al., 2002) we reported results from selected intervals of variable length. Here we will provide a more systematic view on these data, by conducting ANOVAs on 25 ms intervals of these hemispheric differences, as was done above with conventional ERPs, with the factors SOA (83 / 167) and Congruence (congruent, neutral, incongruent). (Representing differences between hemispheres, these data do not include the former third factor, Hemisphere, any more.)

**Figure 6.**
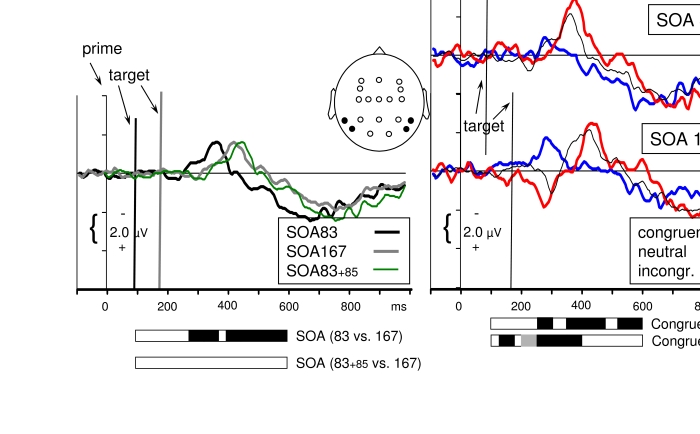
Contralateral-ipsilateral difference waveshapes pooled across the two lateral posterior pairs (|P7-P8|, |PO7-PO8|, i.e., across 2^nd^ and 3^rd^ panels from bottom in Figure 5; positions are indicated by the black dots in the schematic head). The waveshapes in the right panels are the same as in Figure 5 (except for pooling across P and PO and greater scale).The waveshapes in the left panel have been additionally pooled across congruent, neutral, incongruent, to focus on main effects of targets which are obtained by comparing *SOA83* (black) with *SOA167* (grey). The green line in the left panel is the *SOA83* waveshape shifted by 85 ms, to be aligned with the *SOA167* waveshape. As indicated by the additional bar for SOA effects of *SOA83*+85 vs. *SOA167*, these two waveshapes did not differ from each other in the analyzed intervals.Horizontal bars, extending from 100 ms to 600 ms, display significant effects of ANOVAs performed on 25 ms intervals between 100 ms and 600 ms after prime onset. Black shading indicates *p*<.05, gray shading indicates additionally *p*<.06.

**Figure 7.**
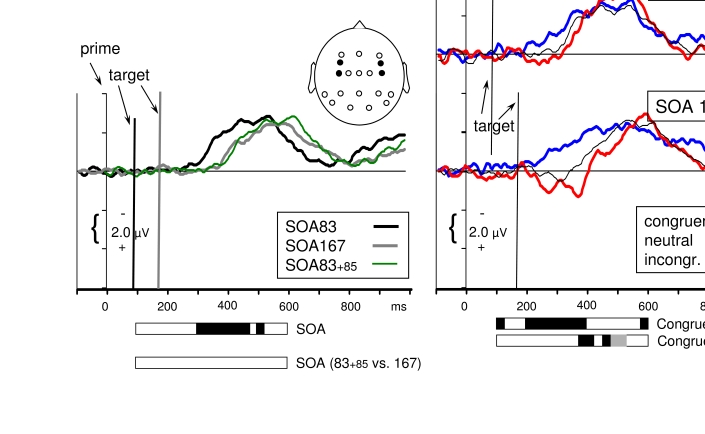
Contralateral-ipsilateral difference waveshapes pooled across the two medio-lateral fronto-central and central sites overlying the (pre-)motor cortex (|FC3-FC4|, |C3’-C4’|, i.e., across 2^nd^ and 4^th^ panels from top in Figure 5; positions are indicated by the black dots in the schematic head). The waveshapes in the right panels are the same as in Figure 5 (except for pooling across FC and C and greater scale). The waveshapes in the left panel have been additionally pooled across congruent, neutral, incongruent, to focus on main effects of targets which are obtained by comparing *SOA83* (black) with *SOA167* (grey). The green line in the left panel is the *SOA83* waveshape shifted by 85 ms, to be aligned with the *SOA167* waveshape. As indicated by the additional bar for SOA effects of *SOA83*+85 vs. *SOA167*, these two waveshapes did not differ from each other in the analyzed intervals.Horizontal bars, extending from 100 ms to 600 ms, display significant effects of ANOVAs performed on 25 ms intervals between 100 ms and 600 ms after prime onset. Black shading indicates *p*<.05, gray shading indicates additionally *p*<.07.

Figure 6 displays with better resolution the potentials recorded from the |PO7-PO8| and the |P7-P8| pairs (pooled across P and PO) and Figure 7 displays the potentials recorded at lateral (pre-)motor pairs |FC3-FC4| and |C3’-C4’| (pooled across FC and C). As in Figures 3 and 4, the left panel displays potentials averaged across congruent, neutral, and incongruent trials, and the right panels display waveshapes separately for congruent, neutral, and incongruent trials. Evidently, these contralateral-ipsilateral difference potentials allow separation of components evoked by targets, by primes, and by congruence of primes and targets, as will be described forthwith.

### Effects in contralateral-ipsilateral differences related to the masking targets

Waveshapes in the left panels of Figures 6 and 7, pooled across primes, display effects of the targets. In the ANOVA, these effects show up as main effects of SOA, since target onset differs by 83 ms between SOAs, and correspondingly target effects are shifted in time. (The green lines in these panels indicate the *SOA83* waveshape shifted by 85 ms, to be aligned with the *SOA167* waveshape. These waveshapes did not differ from each other in the analyzed intervals, indicating that target effects were equal for both SOAs.)

**Posterior sites** (Fig. 6): Well visible is a contralateral negativity, which is the N2pc evoked by the target. It reached its peak at 365 ms with *SOA83* and at 425 ms with *SOA167*, that is, 260-280 ms after target onset. This target-evoked N2pc was reflected by effects of SOA at 280-375 ms (N2pc already starting with *SOA83* but not with *SOA167*) and from 400 ms onwards. This latter long-lasting effect did not only reflect that the target-evoked N2pc still continued at *SOA167* and ended at *SOA83*, but also indicated the onset of a positive shift that started with *SOA83* but not yet with *SOA167*. (This shift probably indicates somatosensory reafference, related to the act of manually responding, Wascher & Wauschkuhn, 1996). Finally, the figure suggests that there was some slight positive peak preceding N2pc at 140 ms after target onset, around 220 ms with *SOA83* and around 300 ms with *SOA167*. While the latter effect might have contributed to the SOA effect around 300 ms, the *SOA83* effect was significant only when neutral primes were considered (see below).

This target-evoked N2pc coincided with the posterior N2 component, which was also clearly distinguished between SOAs (cf. Figure 3 with Figure 6). The present procedure subtracted out all preceding non-lateralized components, thereby providing a stricter isolation of this target-related effect than the N2 did. Perhaps more importantly, as will be reported below, this N2pc was affected by priming in a characteristic way.

**Anterior sites**: At anterior sites, Figure 7 (left panel) displays one obvious target-related effect, which is enhanced negativity contralateral to the target, and at the same time contralateral to the responding hand. Indeed, this component is probably a mixture of response-related activation (“Lateralized Readiness Potential”, LRP, Coles, 1989) and pre-motor attention-related activation (“N2cc”, meaning “N2 central contralateral”, in analogy to N2pc meaning “N2 posterior contralateral”, Praamstra & Oostenveld, 2003). The main effects of SOA 300-475 ms and 500-525 ms reflect the earlier rise of this N2cc-LRP complex with *SOA83* when targets were presented earlier. (The reverse effect, a later decrease of activation with *SOA167*, did not become significant within the analyzed period up until 600 ms.)

This N2cc-LRP complex could not be interpreted as the lateralized portion of some component visible in the conventional ERPs, occurring considerably earlier than the non-lateralized SOA effect (430-525 ms in Figure 4). Importantly, the N2cc-LRP complex was also affected by priming in characteristic ways.

### Effects in contralateral-ipsilateral differences related to the masked primes

Waveshapes in the right panels of Figures 6 and 7 display effects of the primes, separately for both SOAs. In the ANOVA, these effects show up as main effects of Congruence, when equal for both SOAs, and as interactions of Congruence x SOA when different between SOAs. By definition, primes have their relevant shape at the same side as the target when congruent, and at the opposite side when incongruent. Therefore, in the contralateral-ipsilateral differences, potentials directly evoked by primes can be identified as components that are mirror-symmetric, going in opposite directions for congruent and incongruent primes. In addition, there may be indirect effects of primes resulting from their effect on components related to target processing.

**Posterior sites**: The major *direct* signature of the masked primes, reflected by its opposite polarity for congruent and incongruent primes, was the prime-evoked N2pc, evoked almost exclusively by the incompletely masked stimuli with *SOA167*. Indeed, the SOA x Congruence effect, which was significant for 75 ms, at 255-325 ms (and tended to be significant already before, 230-250 ms, *p* = .059), indicated during the entire time span that the simple effect of Congruence was significant for *SOA167* but not for *SOA83* (even though there was also a main effect of Congruence at 255-300 ms). We note that no corresponding effect was visible during this entire time span with *SOA167* in the conventional ERPs (Fig. 3). Additionally, this prime-related effect for *SOA167* overlaps with the early contralateral positivity evoked by targets, best seen by neutrally primed targets (mentioned above in “effects in contralateral-ipsilateral differences related to the masking targets” as well as below in the present chapter).

The major *indirect* effect of these masked stimuli was their priming of the target-evoked N2pc: This N2pc was absent when primes were congruent. This effect was indicated by the interaction of SOA x Congruence (330-400 ms) and by the main effect of Congruence (350-475 ms). At the first interval (330-350 ms) Congruence had its effect with *SOA83* only, evidently because targets were presented earlier with this SOA. In the next interval (355-375 ms) this new effect also started with *SOA167* but remained smaller than with *SOA83* until 400 ms. From 400 ms onwards, the effect was also fully developed with *SOA167*. The effect was larger for incongruent than neutral primes at 380-400 ms with *SOA83* and at 430-450 ms with *SOA167*. We note that this priming effect had a pattern quite different from the priming effect that was visible during this time span in the conventional ERPs (Fig. 3) and that was probably reflecting the temporal delays in response preparation.

In addition to these two conspicuous effects (already described in Jaśkowski et al., 2002, by measurements of selected intervals), other effects of Congruence were found:

An early direct effect of primes, with opposite polarity for congruent and incongruent waveshapes, was indicated by the SOA x Congruence effect at 130-175 ms. Figure 6 suggests a marked tendency with *SOA167* above all, but this did not become significant as a simple effect. What was significant was the difference between the incongruent waveshapes with *SOA167* and with *SOA83*. This difference of polarity between SOAs casts some doubt on the reliability of the effect.

The next, brief SOA x Congruence effect (205-225 ms; gray in Figure 6, because p = .06 only) reflected the contralateral positivity evoked by targets after neutral primes with *SOA83* (about 130 ms after target onset). For *SOA167*, a similar effect can be seen in the neutral waveshape at 280-325 ms (overlapping with the N2pc evoked by the prime), that is, again about 130 ms after target onset.

The final effect of Congruence (530-600 ms, probably further continuing after 600 ms) indicated a temporal delay according to congruence conditions of the late contralateral positive waveshapes. Starting earlier with *SOA83* than with *SOA167*, this effect was probably responsible for part of the Congruence effect with *SOA83* from 400 ms onwards.

**Anterior sites**: The major, obvious effect of primes on the N2cc-LRP complex was that congruent and incongruent waveshapes diverged into different directions from the neutral waveshape. The effect started at 205 ms, which is 50 ms earlier than the prime-evoked N2pc at posterior sites, and continued for almost 200 ms, up until 375 ms, without any measurable difference between the two SOAs. Only at the right margin (380-425 ms) was the effect larger for *SOA167* than for *SOA83* (SOA x Congruence). There was only weak evidence for differences between SOAs at the left margin (205-225 ms, an earlier effect with *SOA83* than with *SOA167*), which did not become significant.

The later intervals of Congruence x SOA effects reflected the larger negative peaks of waveshapes in incongruent trials, 455-525 ms with *SOA83* and later (580-600 ms) with both SOAs.

A very early effect of Congruence (105-125 ms) appeared to reflect a divergence of congruent and incongruent waveshapes with *SOA167* above all, but the simple effect of Congruence with *SOA167* did not become significant.

This latter very early effect, if reliable, would be a direct effect of primes, of course, occurring even before target onset with *SOA167*. In contrast, it is debatable whether the major effect of primes on the N2cc-LRP complex was a direct or an indirect effect. An indirect effect would mean that the prime modified the (pre-)motor activation induced by the target, while a direct effect would mean that the prime directly initiated (pre-)motor activation. In other words, the question is whether the earliest indications of the Congruence effect were initiated by the target or by the preceding prime. If initiated by the target, the onset of the Congruence effect should vary between SOAs by an amount around 83 ms (167-83). This was not the case. True, the Congruence effect was more reliable at 205-225 ms with *SOA83*, being significant in a separate analysis for *SOA83* and not for *SOA167*, but the interaction Congruence x SOA was not significant at this interval, and even so, this would constitute a delay of only 25 ms rather than 83 ms. An additional point in favor of this interpretation is that the onset of the effect for *SOA167*, latest at 230 ms, was only 60 ms after target onset, which appears to be too early to be due to the target. Another criterion for distinguishing between prime- and target-related effects is that the potentials evoked by congruent and incongruent primes should be mirror-symmetric to the baseline if initiated by the prime, at least as long as there is no other target-related activation yet. This symmetry to baseline was the case for *SOA167*, more or less during the entire duration of the Congruence effect (200-400 ms). With *SOA83*, this also seemed to be the case for the early part of the effect, 200-300 ms, after which time-point target-related activation started, which continued to be modulated by the prime-effect. We draw the conclusion that at least the early part of the effect was a direct effect of the primes. The later part of the effect might either constitute a qualitatively different process, namely indirect effects exerted by the prime on target-related motor activation. Alternatively, the waveshape might reflect the parallel existence and addition of two independent activities, namely prime-evoked and target-evoked motor activation.

### Summary of effects on contralateral-ipsilateral differences

There were five major results:

1) A direct effect of the masked primes was their N2pc. This N2pc was evoked with *SOA167* only.

2) Another direct effect of the primes was the early part of motor-related activation. This activation was not statistically different between *SOA83* and *SOA167*.

3) Starting at 205 ms, this motor-related effect of primes (#2.) did not start later than the perception-related effect (#1.), which had its onset at 230 ms. This was also true for earlier effects (which might be unreliable anyway): A motor-related effect was noted at 105-125 ms, a perception-related effect at 130-175 ms.

4) Consecutively, targets evoked their N2pc equally for both SOAs. One major indirect effect of the primes was that this N2pc did not occur after congruent primes. This priming effect on perceptual processing was equal for both SOAs.

5) Targets also evoked motor-related activation. This activation was modulated by the preceding prime-evoked activation (#2.), and thus appeared as an on-line indication of motor priming.

### Summary of comparing effects on contralateral-ipsilateral differences to effects on conventional ERPs

1) The N2pc evoked by primes with *SOA167* ms had no correspondence in its time-range (255-325 ms) in conventional ERPs at posterior sites (Fig. 3). True, the entire P1-N1 complex that preceded this time interval was evoked by prime onset. However, at this relatively late interval, there appeared to be no way of disentangling prime- and target-evoked activity in the conventional ERPs.

2) Likewise, the early part of the N2cc-LRP complex had no correspondence in its time range (200-300 ms) to conventional ERPs at anterior sites (Fig. 4).

3) Thus, in contrast to contralateral-ipsilateral difference potentials, no comparison could be made in conventional ERPs with respect to the earliest time-point of relevance processing.

4) The target-evoked N2pc coincided well in time with the posterior N2 of the conventional ERPs. Both components were affected by prime congruence but the pattern of effects differed. We tentatively concluded that these two components represent different processes.

5) Target-evoked motor-related activation could be clearly delimited in the LRP component of the contra-lateral-ipsilateral difference (Fig. 7, left panel). This was not possible in conventional ERPs. Conversely, the important effect in anterior recordings of the conventional ERP was the N2 evoked by incongruent prime-target sequences, which did not have any correspondence in the contralateral-ipsilateral differences.

Thus, there was hardly any systematic relation between effects on contralateral-ipsilateral differences and effects on conventional ERPs.

### Discussion of effects on contralateral-ipsilateral differences

The five major results in contralateral-ipsilateral differences, as listed above, will now be discussed.

#### 1) N2pc evoked by masked primes at *SOA167*.

 The relevant shape evoked more negativity at the contralateral visual cortex than did the irrelevant shape at its contralateral cortex. We still concur with the interpretation given by Jaśkowski et al. (2002) for this finding, saying that N2pc reflects top-down controlled selection (Eimer, 1996) of the relevant shape: Participants have their relevant shape (diamond or square, depending on the participant) as a template in working memory, to be matched against the stimuli presented left or right. Stimuli matching the template are preferentially processed. N2pc reflects this preference, probably in areas of the ventral stream (Hopf et al., 2006), and indicates by its nature as a contralateral-ipsilateral difference that this preferential processing occurs in the hemisphere that primarily registered the stimulus. The absence of N2pc with unidentifiable primes (SOA of 83 ms) therefore can be taken to suggest that no such selection can take place when stimuli are heavily masked. 

As noted in Jaśkowski et al. (2002) , N2pc thus appears as a correlate of visual awareness (cf. Koivisto, Revonsuo, & Salminen, 2005; Ojanen, Revonsuo, & Sams, 2003, for similar suggestions). However, the relation between N2pc and awareness is apparently not as tight as we would like it to be. First, the average percentage of correct identification of target shapes in the primes was only 59% with *SOA167*, and nevertheless the N2pc was not principally smaller than it usually is for well visible stimuli (e.g., from our lab: van der Lubbe & Verleger, 2002; Wauschkuhn et al., 1998). In line with this, masked stimuli, supposed to be unidentifiable, did evoke N2pc in our later study (Jaśkowski, Skalska, & Verleger, 2003). Furthermore, in Woodman and Luck’s (2003) study, N2pc was reported not to differ between two conditions where identification rates did differ (66% vs. 84%) and even to occur to some extent in those trials where participants erroneously indicated absence of the relevant stimulus. So one might conclude that N2pc does not have any simple relationship to visual awareness. Possibly, the selection process indicated by N2pc is a necessary but insufficient prerequisite for visual awareness.

The selection process indicated by N2pc may be called a process of “attentional” selection. This might simply be considered a pleonasm because paying attention to something entails its selection for processing. Alternatively, this notion might imply that N2pc reflects a “shift of attention toward the location of the relevant shape” (Jaśkowski et al., 2002, p.53). While we cannot exclude that shifts of attention are indeed initiated by masked stimuli, as argued for example by Scharlau (this volume) and Treccani, Umiltà, and Tagliabue (2006) , we do not concur with this definition any more with regard to N2pc because it implies that N2pc reflects the process of shifting rather than the process of selecting. First, it is not clear why the process of shifting should lead to enhancement of EEG activity contralateral to the target of the shift. Control of shift might be a non-lateralized brain function, for example under control of the right parietal lobe. It might only be by selection of the target, which process we relate to N2pc, that the attentional shift gets its lateralized feature. Second, as will be discussed below (3.), this account leads to an unsolved dilemma when trying to explain the lack of N2pc for congruently primed targets.^3^

#### 2) Early motor activation evoked by masked primes.

The relevant shape evoked more negativity at its contralateral (pre-)motor cortex than did the irrelevant shape at its contralateral cortex, starting at 200 ms after prime onset. Above we argued that this activation was directly induced by the masked stimuli rather than being a modulation of motor activity induced by the following target stimulus.

Of much interest, this prime-induced activation was not smaller with *SOA83* than with *SOA167*. This might be considered a type-2 error but, on the other hand, a common ANOVA on the posterior and the anterior contralateral-ipsilateral differences, with Anterior-Posterior as an additional factor, during the intervals indicating the prime-related effects (205-300 ms), yielded a marked interaction of Ant.-Post. x Congruence x SOA at 280-300 ms (*F* = 10.3, *p* = .001), indicating that there was no interaction of Congruence x SOA for the (pre-)motor component (*F* = 1.1, n.s.) in contrast to the clear differentiation of the Congruence effect according to SOA for the N2pc (*F* = 14.1, *p* < .001). Parallel tendencies were noted for the other three analyzed intervals, reaching *p* = .06 at 230-250 ms. These differential effects can be taken to argue against a type-2 error, at least indicating that the difference between SOAs was less at (pre-)motor cortex than at the visual cortex.

 Therefore, these results provide evidence in favour of the claim made by Vorberg, Mattler, Heinecke, Schmidt, and Schwarzbach (2003) on the basis of response-time results, that the effects of stimuli on the motor system are independent of their visibility.

#### 3) Simultaneous onset of perceptual and motor-related effects of masked stimuli

A serial model of effects of masked stimuli on processing would assume that effects on the perceptual system should occur earlier than effects on the motor system, because perceptual analysis should precede motor activation. This was not the case for the indicators of processing that we measured here. Probably, N2pc is the result of a second pass of analysis in the visual system (possibly indicating “recurrent processing”, Lamme, 2003; Verleger & Jaśkowski, 2006) whereas the motor system may be initiated by purely feedforward processing (VanRullen & Thorpe, 2001). At first sight, this fits physiological models of two pathways of visual processing, with the ventral pathway (reflected by N2pc) being responsible for identification, independently of the dorsal pathway that is responsible for organizing actions (Milner & Goodale, 1995). At second sight, one may wonder why no relevance selection is seen by contralateral-ipsilateral differences from dorsal centres of the visual system (e.g., situated in the intraparietal sulcus). But the |P3-P4| recordings that are probably closest to such centres just seem to pick up a mixture, possibly volume-conducted, of posterior and anterior sites, providing no independent contribution. This might be a measurement problem of the present method. Alternatively, it may be speculated that relevance selection on the dorsal pathway mainly occurs in the pre-motor cortex, as indicated by the contralateral-ipsilateral differences, rather than in parietal areas.

#### 4) The prime effect on the target-evoked N2pc

Targets evoked an N2pc. This could be expected from the large number of earlier studies where N2pcs were reported when relevant and irrelevant stimuli were presented symmetrically from fixation (e.g., as quoted in the introduction: Eimer, 1996; Hopf et al., 2006; Luck & Hillyard, 1994; Wascher & Wauschkuhn, 1996; Wauschkuhn et al., 1998). As with the N2pc evoked by the masked stimuli, also the target-evoked N2pc is assumed to reflect top-down controlled selection of the relevant shape and preferential processing for perceiving the stimulus that matches the stored template of the shape.

 The interesting result is the prime effect: The target-evoked N2pc was suppressed after congruent primes, equally for both SOAs. The lack of N2pc with *SOA83* creates a paradox if N2pc is taken to indicate a shift of attention toward the location of the relevant shape (Jaśkowski et al., 2002): With *SOA167*, N2pc is assumed to be suppressed because attention had already been attracted by the relevant shape in the prime, as indicated by the prime-evoked N2pc. But with *SOA83* there is no prime-evoked N2pc, therefore it has to be concluded that attention was not attracted to the relevant shape in the prime, so there is still a need for a shift of attention to that side, so there should be a target-evoked N2pc. We succeeded in circumventing this paradox in Jaśkowski et al. (2002) by assuming that congruent prime-target sequences work as continuing stimulation, enabling participants to identify the relevant shape in the target without any difficulty such that the attentional “shift becomes unnecessary” (Jaśkowski et al., 2002, p.53). This notion, however, implies that N2pc is due to a call for additional resources: Whenever stimuli cannot be identified and more attention is needed, then attention is shifted, evoking N2pc. This model is not well compatible with the presence of N2pc in response to very simple, easily classified stimuli, as in Eimer (1996) , Wauschkuhn et al. (1998) and others. 

Making a new attempt to solve the apparent paradox, we would like to rephrase the results in terms of N2pc indicating selective processing. Accordingly, with *SOA83* there is no preferential processing of the relevant shape in the prime, and with both SOAs there is no preferential processing of the relevant shape in the target if prime-target sequences are congruent. This leads to the statement that preferential processing of the target is hampered with congruent prime-target sequences.

A look at the prime-target sequences depicted in Figure 2 might create the impression that change of the display is responsible for producing the N2pc. There is no such change with congruent sequences (except that target shapes are somewhat larger than prime shapes). Indeed, with neutral sequences, there is an asymmetrical change, on the side of the relevant shape in the target only, whereas shapes remain the same on the side of the irrelevant shape. So this asymmetry of change might be responsible for producing the N2pc. However, with incongruent stimuli, the change is symmetric: There is both a change on the side of the relevant shape in the target (from irrelevant in the prime to relevant in the target) and there is a change on the other side (from relevant in the prime to irrelevant in the target). In spite of this presence of changes on both sides, these incongruent sequences produce an asymmetry of activation: the N2pc. Thus, the presence of change is not sufficient. However, change might be necessary: We may assume that the relevant shape in the target produces an N2pc only if the target display has changed from the prime display. Such change occurs with neutral and with incongruent sequences but not with congruent ones.

 In Jaśkowski et al. (2002) we had interpreted the priming effect on N2pc as a “positive” effect: No extra capacity is needed any more after congruent primes because identification is so easy. The present interpretation implies that priming of N2pc by congruent sequences might rather indicate a “negative”, adverse effect: The visual system cannot clearly select for relevance if no change of objects is perceived. Thereby, target stimuli in congruent sequences would be perceived more diffusely and vaguely. In essence, we propose that the priming effect with congruent sequences is an effect of forward-masking or of repetition blindness (Kanwisher, 1987) or of blindness to response-compatible stimuli (Müsseler & Hommel, 1997). Further studies are needed to corroborate this interpretation. If true, this would be another dissociation between visual processing needed for identification and response-related processing because the priming effect on response processing, to be discussed in the next section, was positive, being helpful for response processing. 

#### 5) The prime effect on target-evoked motor-related activation

Targets evoked the N2cc-LRP complex, reflecting target-related motor activation, equally for both SOAs. Contralateral motor activation during stimulus processing is a trivial finding, having been demonstrated in probably more than hundred studies since Coles (1989) . Of interest were the effects the primes had on this activation. Such effects of masked primes have been demonstrated in a number of studies before (Dehaene et al., 1998; Eimer & Schlaghecken, 1998; Leuthold & Kopp, 1998). The present variation of SOAs between primes and targets enabled us to investigate more closely the nature of this priming effect. We concluded from the timing and amplitude of the early phase of the Congruence effect (200-300 ms) that this was a direct effect of the masked stimuli on motor activation rather than a prime effect of these masked stimuli on activation triggered already by the target. That early phase was discussed above (2.). The later part of the effect is the priming effect, because this is the effect of the masked stimuli on activation triggered by the target. We stated that two alternatives may account for that prime-induced modulation of the target effect. It might either constitute a process qualitatively different from the early phase, namely indirect effects exerted by the prime on target-related motor activation: Facilitation of the target-induced motor activation if the preceding prime had been congruent, impairment of such activation if the preceding prime had been incongruent. Alternatively, the Congruence effect might indicate the continuing existence of prime-induced motor activation, summing with a constant activation induced by the targets. In this latter case, the prime effect might be simply described as the sum of the (possibly decaying) preceding activation induced by the prime and the more recent activation induced by the target.

 This alternative had been discussed by Verleger, Jaśkowski, Aydemir, van der Lubbe, and Groen (2004) with regard to the impairment of target-related activation following a congruent arrow-prime and a separate mask (cf. Jaśkowski & Verleger, this volume). For those data, we concluded that mask-related impairment works by being added to the target-related activation rather than by modifying that activation. By inference, the same might be true here. So the mechanism of motor priming by masked stimuli (both completely and incompletely masked, *SOA83* and *SOA167*) would be an addition of previous activation to target-related activation.

## CONCLUSION

Contralateral-ipsilateral differences of event-related potentials have proven suitable for separating traces of masked stimuli from their priming effects on following masking stimuli. According to these ERP measurements, direct effects of masked stimuli on response preparation do not depend on their discriminability, and their priming effects on processing of the following target stimuli are qualitatively different for stimulus identification and for response preparation.

## Notes

1 There was a *p* = .06 tendency for an interaction of Hemisphere x Congruence x SOA, reflecting a Hemisphere x Congruence interaction with *SOA83*. However, this interaction could not be resolved in ANOVAs on subsets of the data to indicate effects of Congruence but rather reflected a steeper difference between the left and right hemisphere for incongruent than for neutral and congruent primes, on the basis of larger left- than right-hemispheric N1 in all three cases. Reasons for this effect are unclear and beyond the scope of this contribution.

2 It might be argued that the P1-N1 complex evoked by the targets is the well visible sequence of peaks at about 300 ms and 350 ms with *SOA83* and at about 350 ms and 420 ms with *SOA167*. That is, what we called the target-evoked N2 (see below, at “later posterior effects”) would actually be the target-evoked N1. This suggestion is not very plausible, for two reasons. First, P1 and N1 would be delayed in this case. To elaborate: the positive peak reached its maximum at 220 ms with *SOA83* (300-83 ms) and at 180 ms with *SOA167* (350-167 ms), which is rather late for P1, and the negative peak reached its maximum at 260 ms approximately (350-83 ms and 420-167 ms), which is rather late for N1. Second, the positive peak at *SOA83* did not deviate from the *SOA167* waveshape. Were it the P1-component evoked by the target it should become more positive than the *SOA167* waveshape. So we conclude that the downturn towards positivity common to both SOAs is the P2 component evoked by the prime-target complex. This downturn is terminated by the target-evoked N2 component, earlier with *SOA83* than with *SOA167*.

3 We acknowledge the doubts and objections raised with regard to this problem by Rob van der Lubbe, our coauthor of Jaśkowski et al. (2002), at the time of our writing that manuscript.

## Appendix: Experimental methods

### Participants

Seventeen students of the University of Lübeck participated with payment. Four of these participants were excluded because their discrimination of primes was above chance (≥ 60%) with the short stimulus onset asynchrony (SOA). An additional participant was excluded because his EEG included too many artifacts, leaving data from 12 participants for analysis.

### Stimuli and procedure

Participants looked at a screen from a distance of 1.2 m, sitting in a darkened chamber. The screen background was white, and all stimuli were presented in black. Each trial started with a warning signal, serving as a fixation aid: Four points appeared 1.5° above, below, left and right from screen center, moved inward for 0.9 s, and then “crystallized” to form a fixation cross (0.35° x 0.35°) that remained on during the trial. After 100 ms, the pair of prime stimuli was presented for 17 ms, followed by the pair of main stimuli, presented for 100 ms. SOA between the prime and main stimuli was either 83 ms or 167 ms, in random sequence across trials. The intertrial interval was 2.5 s.

These stimuli were adapted from Klotz and Neumann (1999) : The main stimuli were a rectangle and a diamond, simultaneously presented left and right from fixation (centered 1.5° from fixation), randomly changing sides over trials (Fig. 2). For each participant, either the rectangle or the diamond was defined as relevant. The rectangle was 0.75° wide and 1° tall; the diamond was the rectangle rotated by 45°. The preceding pair of prime stimuli also consisted of rectangles or diamonds, somewhat smaller but centered at the same locations as the main stimuli and fitting within their inner contours (0.6° x 0.8° for the rectangle; the diamond was again rotated by 45°). The pair of primes consisted of either two irrelevant shapes (neutral condition, 50% of trials) or one relevant and one irrelevant shape, with the relevant shape being positioned at the same location as in the main pair (congruent condition) or on the opposite side (incongruent condition).

The experiment comprised two parts, each consisting of 432 trials (108 neutral, 54 congruent, and 54 incongruent trials with each SOA). In the choice response part, participants had to press the left or right response key depending on the side of the relevant shape in the main stimulus. In the signal detection part (always performed afterward, so dark adaptation would be optimal), participants had to press the left or right response key (balanced over participants) depending on whether they believed that the relevant shape had occurred in the prime stimulus or not. Correct and incorrect responses led to 5 cents gained and lost, respectively. Most participants had some success when the SOA was 167 ms, so this reward system amounted to continuous partial reinforcement, thus providing some motivation. The percentage of correct responses was calculated separately for each SOA and amounted to 51% on average for *SOA83* (range 43% to 57%) and to 59% for *SOA167* (range 45% to 76%) for the participants included in the data analysis. The four participants rejected from the analysis because of too good discrimination had 60% – 65% success with *SOA83*.

### Data recording and preprocessing

Manual responses were measured, in analogy to the EEG, as continuous signals from force-sensitive keys, with a response being counted when the force exceeded 2 Newtons.

The EEG was recorded during the choice-response task with Ag/AgCl electrodes, with the tip of the nose as reference, and was amplified from 0.03 to 35 Hz. Intervals from 100 ms before prime onset until 1000 ms afterwards were stored on disk with a sampling rate of 200 Hz (1 data point every 5 ms). Transmission of blink potentials into the EEG were removed by linear regression from vertical EOG to EEG, all other artifacts, as well as incorrect responses, led to rejection of the trial from averaging. Six averages were formed, separately for congruent, neutral, and incongruent trials with each of the two SOAs. Furthermore, to obtain contralateral-ipsilateral differences, the differences between the EEGs contralateral and ipsilateral to the relevant shape in the target stimulus were determined for each symmetrical pair of electrodes. Separate averages were calculated for trials with the relevant shape on the left and trials with the relevant shape on the right, and these averages were then averaged together. Because the shape (diamond or rectangle) that was relevant varied across participants, this contralateral-ipsilateral difference is balanced with respect to the particular shapes used and only reflects stimulus relevance.
